# Investigation of the Level and Factors Influencing Emergency Department Nurses Fatigue: A Case Study of the Saudi Arabian Context

**DOI:** 10.3390/healthcare10071294

**Published:** 2022-07-13

**Authors:** Bushra Alshammari, Albandry AlEnazy, Farhan Alshammari, Norah Madkhali, Mahmoud Al-Masaeed

**Affiliations:** 1 Medical Surgical Nursing Department, College of Nursing, University of Hail, Hail 2440, Saudi Arabia; 2 Buraidah Central Hospital, Al-Qassim 52361, Saudi Arabia; 3 Department of Pharmaceutics, College of Pharmacy, University of Hail, Hail 2440, Saudi Arabia; frh.alshammari@uoh.edu.sa; 4 Department of Nursing, College of Nursing, Jazan University, Jazan 45142, Saudi Arabia; namadkhali@jazanu.edu.sa; 5 Faculty of Health and Medicine, University of Newcastle, Callaghan 2308, Australia; mahmoud.almasaeed@uon.edu.au

**Keywords:** emergency nurses, fatigue, Saudi Arabia, predictors, impact

## Abstract

***Background***: Work-related fatigue is a common health problem among nurses which can affect their performance and decision making. ***Significance and Aim***: The study explores the levels of fatigue and its associated factors among emergency department (ED) nurses in Saudi Arabia. ***Methods***: The study was developed through a cross-sectional quantitative study design. This included the collection of primary quantitative data with a questionnaire prepared and published on REDCap. The study questionnaire was adapted from two tools, namely the OFER 15 and the Copenhagen II tools, respectively. ***Results***: The study established that the Saudi Arabian ED nurses have high acute fatigue (OFER 15 score = 81.11), moderate-high chronic fatigue (OFER 15 score = 74.17), and a high inter-shift recovery index (OFER 15 score = 78.01). In terms of the predictor factors, the study established that for the demographic factors, gender has an impact on chronic and acute fatigue, while work experience impacted acute fatigue and the number of dependents impacted on inter-shift recovery index. On the psycho-social factors, chronic fatigue is influenced by emotional demand (which is a variable used to evaluate the levels to which the nurse is invested, gaining education/skills thus increases job satisfaction) (−0.289), influence at work (−0.310), commitment at the workplace (0.376), rewards (−0.187), stress (0.420), and burnout (0.293), respectively. Acute fatigue is influenced by the emotional demands (0.336), role clarity (−0.128), and the nurses’ well-being and health (−0.034). Finally, the inter-shift recovery index is influenced by the ED nurses’ burnout levels (−0.877). ***Conclusions***: The study indicates a high level of nursing fatigue among the Saudi Arabian ED nurses.

## 1. Introduction

Fatigue is a state of tiredness, exhaustion, and a decline in energy to execute and perform one’s expected functions [1,2]. Fatigue is often characterised by the strong desire to rest and sleep. When people and individuals are fatigued, they are unable to perform their duties and responsibilities to their optimum and often feel sleepy, have less concentration, and often have a low productivity index [2,3]. The effects of fatigue on staff, including the healthcare workers, are diverse. They range from personal to negative professional implications. At an individual level, high levels of fatigue cause ill health and are often associated with mental and psychological ill-health [4,5]. Individuals experiencing prolonged episodes of fatigue are faced with health risks such as stress, anxiety, and depression. Additionally, physical fatigue leads to health complications such as back pains, headaches, and joint pain and discomforts [6,7]. At a professional level, fatigue is associated with declining productivity levels, as well as demotivation. For the healthcare industry, a high fatigue index is associated with low empathy among the healthcare providers, low productivity, lack of concentration, and the risk of medical errors [8,9]. These are risks that are likely to negatively impact emergency nurses. 

Emergency nurses have a social and unique responsibility to take care of the emergency unit patients [10,11]. The patients seeking emergency nurses’ care are often in a critical state of their life and often unable to respond or execute some of the basic duties and responsibilities [10,11]. As a result, they need the absolute care and support of the nurses to help them with the basic functions such as bathing, eating, and taking their medications. This means that the emergency nurses often face higher tasks and responsibilities index on their patients. The additional responsibilities and need for care by the patients expose the nurses to the risk of a higher fatigue index. Additionally, the nurses attend to patients whose conditions are often unstable [12,13]. This means that they need to constantly monitor the patients and their conditions. A timely response to any condition changes is critical and has the effect of potentially saving the patients’ lives. Hence, the emergency nurses need to be extra alert to their context so as to respond quickly and with optimum professionalism to any emerging emergency cases in the facility [14,15]. A contextual analysis of the work-related needs of the emergency nurses indicates a high likelihood and an exposure to nursing fatigue for this nursing category. The findings serve as a validation for the need to explore the levels in the Saudi Arabian context. In Saudi Arabia, a significant proportion of the nurses are foreigners. However, existing data demonstrates that figures and levels for both the national and foreign nurses is relatively the same. This is based on the occupational cause of fatigue investigated in the developed study [13,16]. In addition, ED nurses in the Middle East and in KSA are predominantly female, with a lower proportion of them as male. This is due to the traditional culture and perception of the profession as feminine. However, this is gradually shifting, and more male ED nurses are taking up the profession [12,13]. Some of the key factors identified in the literature as impacting ED nurses’ fatigue levels include emotional demands (the level of skills and dedication towards career growth to realise satisfaction), influence at work, commitment at the workplace, rewards, stress, burnout, role clarity, and the nurses’ well-being and health.

A preliminary analysis of the existing studies demonstrates that only a few researchers studied work-related fatigue among emergency nurses [16,17,18]. In particular, in the Middle East, including Saudi Arabia, the studies investigating the level of healthcare workers' fatigue levels, especially the emergency nurses, are very few. This is especially with a high gap in primary-based research studies on the topic. The findings of this study could be baseline information for healthcare professionals in order to develop appropriate intervention programs and strategies among ED nurses to minimise work-related fatigue. Some of the key areas of predictors of work-related fatigue among ED nurses in KSA evaluated in the study include an examination of the relationship between the socio-demographic factors, psycho-social work stressors, and work-related fatigue among emergency nursing staff. Therefore, this study investigates the existing fatigue levels among emergency nurses in Saudi Arabia and the fatigue influencing and causative factors. This study is unique and has originality in the sense that it focuses on the Middle East ED nurses who have not been investigated satisfactorily in the past. As such, it brings in new literature on the similarity or differences on factors influencing ED nurses’ fatigue in the KSA and Middle East as compared to the existing global literature.

## 2. Materials and Methods

### 2.1. Design

A cross-sectional, descriptive correlational design was used to conduct this study. Data was collected over a three-month period. This was the period after the COVID-19 surge had declined in Saudi Arabia and the ED nurses were back to their regular shifts and responsibilities. The adoption of a cross-sectional design means that the researcher would only collect the fatigue and influencing factors data only once and then analyse the findings. The rationale for this was based on two fundamental factors. First, the examined study variable of fatigue is a relatively stable variable and thus not likely to change in the short-term period. It means that the experienced levels of fatigue only change gradually over time, thus making the collecting of data at a single point within a long-term period such as a year or an estimated two years enough for the developed study. As such, it meant that since fatigue levels were expected to change over an estimated minimum one-year period, the study could not collect longitudinal data as this would imply collecting data at intervals of one year, which was outside the anticipated study time limits. Ethical approval was obtained from the ethical committees in the general directorate of health affairs in Al-Qassim city and from the Hail Health cluster in Hail city where the study was conducted.

### 2.2. Data Collection

The data collection process was based on targeting eight of the leading emergency hospitals in two cities in Saudi Arabia, namely Hail and Al-Qassim. This includes Buraidah Central Hospital, King Fahad Specialist Hospital, King Saud hospital, Alrass General Hospital and King Salman Specialist, King Khaled hospital, and the Maternity and Children Hospital in Hail and Al-Qassim city.

After ethical approval was obtained, the targeted hospitals were reached through formal email communication with the administration and the nursing in-charge or manager. The head of nursing distributed the flyers to their emergency nursing staff members. The flyer contained a detailed explanation of the study including its aim and objectives. It also listed the criteria required to participate in the study. Eligible participants must be registered nurses, with more than one year experience of working in the emergency department. The nurses who were on leave but remained in inactive employment were also included in the study. Any emergency nurses who had retired and thus not in active employment were excluded from the study.

The researcher posted and published the questionnaire on Research Electronic Data Capture (REDCap). This is a secure online platform allowing for the publishing of clinical research questionnaires and data [19,20].

### 2.3. Instruments

A self-administered structured questionnaire was used and consists of the following instruments: basic socio-demographic data questionnaire, Occupational Fatigue Exhaustion/Recovery Scale (OFER15), and Copenhagen Psychosocial Questionnaire version Two (COPSOQ II). 

#### 2.3.1. Occupational Fatigue Exhaustion/Recovery Scale (OFER15)

The OFER15 tool was developed by Winwood et al. (2006) [21]. It comprises 15 items distributed on three subscales (acute fatigue, chronic fatigue, and inter-shift (recovery). Each subscale consists of five items as follows. Each item is rated with seven-points on the Likert scale from “0” (strongly disagree) to “6” (strongly agree). The items that were numbered with 9, 10, 11, 13, and 15 should be scored reversely. The OFFER 15 manual states that when computing for the variables, OFER-CF = sum (item 1–5 scores)/30 × 100, OFER-AF = sum (item 6–10 scores)/30 × 100, and OFER-IR (or OFER-PF) = sum (item 11–15 scores)/30 × 100. The sum of each subscale is divided by 30 and multiplied by 100 to produce comparable scores between 0 and 100 [21]. The OFER’s scale demonstrated high constructs and face validity [22]. 

#### 2.3.2. Copenhagen Psychosocial Questionnaire Version Two (COPSOQ II)

It was developed by the National Research Centre for the Working Environment, Denmark [23]. It has three versions or forms: long (128 questions), medium (87 questions), and short form (40 questions). The short version is the most appropriate for the workplaces (Pejtersen et al., 2010 [23]). The COPSOQ II short version measured 23 psycho-social factors with a total of 40 questions. Most of the questions were rated based on five response options except job satisfaction and work family conflict, which will rate on four options. The first five response options were: always = 4, often = 3, sometimes = 2, seldom = 1, and never/hardly ever = 0. The scoring system of COPSOQ II is very simple. For the questions with 5 response options, the scores are rated as 0, 1, 2, 3, and 4. To measure the total score of each factor, we can add the two scores of two questions, and it will be within 0–8. 

### 2.4. Data Analysis 

The final step in the study was data analysis and presentation. The data analysis stage was a sequential process. Once the findings had been recorded on REDCap, the researcher downloaded an excel file version of the data. The version was then cleaned up to eliminate and remove any responses done halfway and any of the personal identifier details that could have been captured through the system [24,25]. Once the data was cleaned up, the researcher saved the excel file as the original data file for future verification of data analysis. The file than was converted into an SPSS file version [26]. The analysis was based on the OFER 15 manual scoring guidelines. 

## 3. Results

### 3.1. Socio-Demographic Characteristics of the Study Sample

Overall, the sample base distribution and representation were equitable enough for the study to proceed with the analysis with a sample base of 125 respondents. This is as summarised in the table below. A summary of the findings is illustrated in Table 1 below.

### 3.2. OFER 15 Tool Analysis

The findings are illustrated in Table 2.

From the above table findings, the respondents had a moderate/high score of 72.67 on chronic fatigue. This was in addition to a high score of 80.43 on acute fatigue and a high score of 77.49 on inter-shift recovery fatigue.

### 3.3. Copenhagen Variables Analysis

The response scores varied from a minimum score of 1 to a maximum of 5 In presenting the findings, the possible maximum score was indicated to allow for the mean and median values computation. The descriptive findings demonstrating the mean, standard deviation, and skewness for the variables are demonstrated in Table 3 below.

A summary of the findings above indicates that all the variables examined under the Copenhagen II tool as causative factors for fatigue among nurses had a significant index and levels among the nurses, especially for all the upper ranges of the mean values considering the standard deviations, with an average upper limit average mean range above the median value of 2.5. This meant that the nurses considered them as important, thus creating validation for evaluating the relationship and link between the nurses′ fatigue level and the variables.

### 3.4. Relationship between Nurses Fatigue and Nurses Socio-Demographics

The study examined the relationship between the nurses' fatigue levels (as analysed through the OFER 15 tool) and their socio-demographic variables (presented in Table 1 above). The findings were analysed for statistical correlation with a significance level of 0.05. Additionally, those with a statistical significance of 0.02 were also noted and marked. The correlation scores run from 0 to 1 with a score close to zero (0) indicates a weak correlation and a score close to 1 indicates a high and rising relationship/correlation between variables. The findings are presented in the table below with r representing the correlation value and p representing the sigma/significance value. The findings are illustrated in Table 4 below.

The above findings indicate that the nurses’ gender is positively correlated to the nurses’ chronic and acute fatigue. In relationship with chronic fatigue, it had an r value of 0.404 at *p* < 0.01 significance level and 0.324 for acute fatigue at sigma value of *p* < 0.01. The correlation scores are a fair correlation index. This means that the nurses’ gender has a role and influence on the nurses’ fatigue levels. Equally, the nurses’ gender related to their acute fatigue with an r value of 0.324 at a significance value of *p* < 0.01. The positive correlation means that as the gender shifts from male to female nurses, the level of fatigue rises considerably. This is to mean that the female nurses have a higher fatigue index than their male peers. 

In addition, there was a negative correlation between experience and acute fatigue among nurses. This meant that the nurses had a lower acute fatigue index as their experience years increased. This was at an r value of −0.028. This is a weak negative correlation between the variables. For the inter-shift recovery index, the analysis established a significant negative correlation between recovery and the number of dependents. This meant that as the nurses had more dependents, their ability to recover from acute fatigue during their rest periods before the next shift declined considerably. This was at an r value of −0.061. This is a fair negative correlation meaning that the number of dependents that the nurses, especially the female nurses, have to care for has a direct impact on their available resting time while off work.

### 3.5. Relationship between Nurses Fatigue and Socio-Cultural Variables

This section provides a relationship analysis between the nurses’ fatigue levels (as analysed through the OFER 15 tool) and the Copenhagen II variables. In examining the relationship, a correlation analysis was developed. The findings are illustrated in Table 5 below.

The findings indicated that the nurses′ chronic fatigue was impacted by the emotional demands (−0.289), influence and work (−0.310), commitment at the workplace (0.376), burnout (0.293), and stress (0.420), respectively. On the one hand, there is a positive correlation between the commitment to the workplace, burnout, and stress variables. On the other hand, there is a negative correlation between chronic fatigue and the emotional demands, influence and work, and the rewards (recognition) variables. This is because a rise in these variables, such as earnings, means more opportunities for managing fatigue, such as through proper dieting, exercise payment, and programs, among others. (The earnings are represented in Saudi Arabian Riyal (SAR) where the average monthly national earnings in Saudi Arabia is 4230 SAR). Equally, a rise in work influence indicates inclusion in decision making and participation in nursing activities planning, thus reducing fatigue among the nurses. 

For the nurses’ acute fatigue, the findings indicate a correlation with emotional demands (−0.336), role clarity (−0.128), and well-being and health (−0.034). For all the variables, the correlation was negative. The correlation between acute fatigue and role clarity is based on the understanding that with clear roles, there is minimal confusion, minimal conflicts among nurses due to duties execution, and resulting in the elimination of a risk of duplication of efforts. A 0.128 correlation indicates a weak negative relationship between the variables. Equally, the nurses’ well-being and health has a direct influence on their fatigue levels, as when healthy, they execute their responsibilities optimally as opposed to when they are unwell. This equally applies in the emotional demands variable, where if the nurses are emotionally involved in their work, the fatigue levels are reduced. Finally, the analysis of the finding indicated a strong positive correlation between inter-shift recovery and the nurses’ burnout levels at 0.877. This meant that a rise in the explore and level of nurses’ burnout, which in the context implies the reduction in energy due to long, frequent, and repetitive shifts, leads to a rise in the nurses' fatigue levels.

## 4. Discussion

### 4.1. Fatigue Levels

One of the study objectives was an evaluation of the existing fatigue levels among the Saudi Arabia emergency nurses. The findings indicate significant fatigue levels with a moderate-high chronic fatigue index and a high acute fatigue index. The existence of significant fatigue levels among Saudi Arabia nurses reflects on the prevailing significant levels for ED nurses globally. For example, previous studies by Barker and Nussbaum [27] and Woo, Ho, Tang, and Tam [28], while focusing on the global context, demonstrated that there exists a high fatigue index among nurses. This was an argument adopted and advanced by other players focusing on the western and the Middle East regions, respectively.

In particular, studies such as Kim and Choi [29] and Jarrad and Hammad [30] indicated that the exposure and risk to high fatigue levels was significantly higher for the emergency nurse. This was attributed to their unique nature and direction of work. Thus, the findings in the Saudi context are a reflection of the existing environment approach. While as a majority of the studies in the western countries indicated moderate and moderate-high levels of fatigue, the levels of fatigue in Saudi Arabian emergency nurses were relatively higher [31,32]. On the nurses’ recovery index, it was evident that the nurses have a high inter-shift recovery index, demonstrating a rationale for the lower index for chronic as compared to the higher index for acute fatigue. This is to imply that the highest source of fatigue for the Saudi Arabia emergency nurses is based on their shifts, as they are more fatigued during their shift periods with acute fatigue than in the long run period as demonstrated by a moderately high chronic fatigue index.

### 4.2. Nurses Demographic Factors and Nursing Fatigue

An additional study objective was to investigate and explore the predictors and causative factors for nurses’ fatigue among emergency nurses in Saudi Arabia. The study findings demonstrate that the nurses’ gender was directly related and impacted the nurses’ chronic fatigue levels. This was to mean that the chronic fatigue levels between the male and the female nurses differed. A further in-depth analysis indicated that the female emergency nurses had a higher exposure and level of chronic fatigue as opposed and compared to their male peers.

The findings that the female nurses had higher chronic fatigue were similar to other related literature in the Middle East context. For example, the Kim and Choi [29] and Chemali et al. [33] studies applied the theory of the social role in defining the role and responsibilities based on gendered roles. The studies indicated that the female gender was entrusted with additional duties and responsibilities such as domestic care for their families alongside their professional responsibilities. Thus, while the male gender nurses have enough rest time to recover from fatigue, the female nurses have lower recovery and time to rest, thus increasing their exposure to chronic fatigue. This is a variable in the study that was further illustrated in the relationship between gender and acute fatigue levels. The acute fatigue variables could be explained by the prevailing culture and norms in Saudi Arabia. One of the values as illustrated in the Ledoux [34], Chemali et al. [33], and Al-Masaeed, O’Brien, Rasdi, and Alqudah [35] studies were the belief and demand that same-gender nurses treat and care for same-gender patients. As such, the female nurses have to take care of the female patients. Thus, an imbalance of the patients' and nurses' gender exposes the nurses to fatigue based on their gender. Finally, the findings demonstrated a negative relationship between inter-shift recovery index and the nurses’ number of dependents. This was to mean that a higher number of dependents led to a lower inter-shift recovery index. This implies that with more dependents to care for at home, the nurses had minimal rest periods and thus low inter-shift recovery index. This is a variable further explained by the Al-Masaeed, O’Brien, Rasdi, and Alqudah [35] study. They argued that this has a higher impact index on female nurses, who take up the domestic care providers for their families and children when off the shift.

### 4.3. Phsycho-Social Factors and Nursing Fatigue

Regarding chronic fatigue, the findings indicated that there was a significant correlation between emotional demands, influence at work, commitment at the workplace, rewards and recognition, burnout, and stress, respectively. The findings indicate a negative relationship between chronic fatigue and emotional demands, influence and work, and rewards. This is to mean that as these variables increase, there is a reduced exposure and risk for the nurses’ chronic fatigue. The emotional demands raise means that the work is highly engaging and emotionally demanding, thus providing an opportunity for career growth which lowers the nurses’ risk of chronic fatigue [22,36]. Equally, the nurses’ influence at the workplace has an impact of elevating their motivation and dedication to their professional work since they feel a part of the decision making in the workplace. Furthermore, a negative relationship to rewards and recognition means that as the nurses are compensated for their services with an equal and better reward, they are satisfied and thus have a lower chronic fatigue index [37,38].

In addition, the findings indicated a positive relationship between stress, burnout, and commitment in the workplace variables on the nurses’ chronic fatigue. This is to imply that as the nurses experience a higher stress and burnout index, both in the workplace and at home, their exposure to long-term fatigue significantly rises. Equally, as commitment rises, it means they spend more hours in their workplace and dedicated to service delivery, thus exposing them to the risk of long-term chronic fatigue [37,38].

Moreover, the findings indicated a relationship between the nurse acute fatigue and their emotional demands, health and well-being, as well as the role clarity index in the workplace. For emotional demands, the engagement of the nurses’ emotions in the workplace means that they are invested and educated about their responsibilities, thus making their shift work enjoyable and reducing their exposure to acute fatigue. This is a finding also evident with the role of the clarity variable. The more the roles are clear, the less the confusion and duplication of efforts. The nurses are clear on what is expected of them, thus reducing the risks of double tasks execution as well as confusion when executing their responsibilities during a shift period. Finally, the nurses’ well-being directly influences their acute fatigue levels [39,40]. When the nurses are in good health, they have enough physical and emotional energy to handle their shift responsibilities, thus lowering fatigue levels. On the contrary, if they are unwell, they have less energy both physically and emotionally, thus increasing their exposure to acute fatigue. The context of good health and well-being is categorised and evaluated on both the physical and emotional dimensions [38,40].

The final finding was on the psycho-social variables impacting the nurses' inter-shift recovery index. The findings demonstrated that there were significant negative correlations between the nurses’ inter-shift recovery index and their burnout levels. This was to mean that the more the nurses were experiencing a burnout, the lower their inter-shift recovery ability.

## 5. Implications

The study findings indicate that the ED nurses′ fatigue in the KSA and the larger Middle East region is slightly higher than the fatigue levels in the developed markets such as Europe and the USA. Thus, it demonstrates the need for fatigue mitigation and management strategies. Based on the findings, the study recommends that future studies should target interventions to reduce fatigue among ER nurses, taking into consideration the factors that influence fatigue.

## 6. Conclusions

In summary, the study indicates a high level of nursing fatigue among the Saudi Arabian ED nurses. It is evident that the nurses’ socio-demographic factor, such as gender, has an impact and influence on the nurses’ fatigue levels. Equally, it is demonstrated that a majority of the Copenhagen II variables have an impact and influence on the ED nurses’ fatigue levels. With the above understanding, it is evident that there is a need for practitioners and regulators in the Saudi Arabian healthcare sector to develop strategies and policies to help curb and reduce the ED nurses’ fatigue levels. 

## Figures and Tables

**Table 1:** Nurse socio-demographic variables.

Variable	Sub-Scale	Frequency	Percentage
Gender	Male	26	20.8%
	Female	99	79.2%
Age	20–29 years Mean Age 27.2	66	52.8%
	30–39 years SD 2.3	48	38.4%
	40–49 years	7	5.6%
	50 and above years	4	3.2%
Education Level	Diploma	15	12%
	Bachelor’s Degree	99	79.2%
	Master’s Degree	11	8.8%
	Doctorate Degree	0	
Marital Status	Single/Divorce/Widowed/Separated	65	52%
	Married	60	48%
Number of Dependents	None	66	52.8%
	Less than 3	48	38.4%
	3 and Above	11	8.8%
Career Rank	Supervisor	0	
	Head Nurse	15	12%
	In Charge Nurse	9	7.2%
	Staff Nurse	101	80.8%
Year of Experience	1–5 years	86	68.42%
	6–10	15	12.28%
	11–15	11	8.77%
	16–20	13	10.53%
Weekly working Hours	Below 40 h	3	2.4%
	40–45 h	39	8.8%
	46–50 h	83	88.8%
Monthly Income	Less than 6000 SAR	78	62.4%
	From 6000–10,000 SAR	25	20%
	More than 10,000 SAR	22	17.6%
Shift Worked	Day 12 h	18	14.04%
	Night 12 h	15	12.3%
	Day 8 h	72	57.9%
	Night 8 h	31	24.6%
	Evening 8 h	61	49.1%

**Table 2:** OFER 15 fatigue analysis.

		Descriptive Statistics				
	N	Minimum	Maximum	Sum	Mean	Std. Deviation
Chronic Fatigue	125	26.67	103.33	9082.67	72.6613	21.36146
Acute Fatigue	125	33.33	100.00	10,053.33	80.4267	15.11590
Inter-shift Recovery	125	33.33	100.00	9686.67	77.4933	13.80001

**Table 3:** Descriptive findings demonstrating the mean, SD, and skewness for the variables.

Descriptive Statistics						
	Minimum	Maximum	Mean	Std. Deviation	Skewness	
	Statistic	Statistic	Statistic	Statistic	Statistic	Std. Error
Quantitative Demands	1.75	4.25	2.7460	0.50997	0.555	0.316
Emotional Demands	1.00	5.00	2.8000	1.05111	0.134	0.316
Influence At work	1.00	5.00	2.5560	0.98213	0.523	0.316
Possibilities for development	1.00	4.00	2.3600	0.78443	−0.191	0.319
Meaning of work	1.00	5.00	2.0920	0.91666	0.758	0.316
Commitment at the workplace	1.00	4.00	2.3000	0.98579	0.148	0.316
Predictability	1.00	5.00	2.4960	0.96615	0.183	0.316
Rewards (Recognision)	1.00	5.00	2.5520	1.04885	0.403	0.316
Role clarity	1.00	4.00	2.3080	0.82253	0.117	0.316
Role of Leadership	1.00	5.00	2.5060	0.91662	0.319	0.316
Satisfaction with work	1.00	4.00	2.5280	0.61978	0.239	0.316
Scale for Justice and Respect	1.00	5.00	2.5840	1.04097	0.299	0.316
Well-being and Health	1.00	5.00	2.6480	0.95119	−0.048	0.316
Burnout	1.00	5.00	3.2720	0.84363	−0.420	0.316
Stress	1.00	5.00	3.2600	0.98087	−0.269	0.316
Offensive Behaviour	1.75	5.00	4.0740	1.01154	−0.755	0.316
Valid N (listwise)						

**Table 4:** Relationship between fatigue and nurses’ socio-demographic variables.

Factors	Chronic Fatigue		Acute Fatigue		Inter-Shift Recovery Index	
	r	*p*	r	*p*	r	*p*
Gender	0.404 **	0.000	0.324 *	0.000	0.220	0.241 r = 0.007
Age	0.043	0.748	0.072	0.594	−0.007	0.957
Education Level	0.104	0.443	−0.003	0.982	−0.122	0.365
Marital Status	0.121	0.372	0.131	0.330	0.034	0.799
Number of Dependents	−0.019	0.890	0.080	0.552	−0.061	0.042
Career Rank	−0.090	0.504	−0.041	0.764	0.116	0.390
Year of Experience	−0.078	0.562	−0.028 *	0.048	−0.107	0.428
Weekly working Hours	0.014	0.920	0.007	0.960	0.231	0.029

* Significance at 0.05, ** Significance at 0.02.

**Table 5:** Relationship between fatigue and the Copenhagen ii variables.

Factors	Chronic Fatigue		Acute Fatigue		Inter-Shift Recovery Index	
	R	*p*	R	*p*	R	*p*
Quantitative Demands	−0.047	0.727	−0.024	0.860	−0.015	0.911
Emotional Demands	−0.289 *	0.029	−0.336 *	0.010	−0.111	0.412
Influence and Work	−0.310 *	0.019	−0.235	0.078	0.292	0.027
Possibilities for development	−0.148	0.275	−0.115	0.399	−0.178	0.190
Meaning of work	0.062	0.647	0.072	0.595	0.090	0.044
Commitment at the workplace	0.376 **	0.004	0.091	0.501	0.299	0.024
Predictability	0.069	0.608	−0.068	0.613	0.099	0.465
Rewards (recognition)	−0.187 *	0.044	−0.067	0.622	0.096	0.0.47
Role clarity	0.098	0.469	−0.128 *	0.034	0.105	0.436
Role of leadership	−0.212	0.014	−0.083	0.539	0.071	0.599
Satisfaction with work	−0.001	0.994	−0.018	0.893	0.042	0.757
Scale for justice and respect	0.251	0.059	−0.044	0.748	0.023	0.865
Wellbeing and health	0.044	0.743	−0.034 *	0.032	−0.055	0.686
Burnout	0.293 *	0.027	−0.219	0.102	−0.877 *	0.520
Stress	0.420 **	0.001	−0.191	0.154	−0.226	0.091
Offensive Behaviour	−0.029	0.829	−0.086	0.525	0.009	0.950

* Significance at 0.05, ** Significance at 0.02.

## Data Availability

Not applicable.
